# Dementia Care Aware: Building Primary Care Capacity for Early Detection of Dementia in California

**DOI:** 10.1002/alz70860_102210

**Published:** 2025-12-23

**Authors:** Anna H Chodos

**Affiliations:** ^1^ University of California San Francisco School of Medicine, San Francisco, CA, USA

## Abstract

California's Medicaid program, Medi‐Cal, expanded its benefits in July 2022 for older members to include an annual screening for dementia. Providers using this benefit were required to take an accompanying training and therefore funded a program called Dementia Care Aware to provide the training and disseminate it to eligible primary care clinicians across the state. Led by the University of California, San Francisco (UCSF), Dementia Care Aware initially partnered with other UC campuses and dementia‐focused community‐based organizations to realize the goal of reaching primary care in all of California's 58 counties. As of October 2024, Dementia Care Aware continues in a partnership between the West Health Institute and UCSF. Attendees will learn the key accomplishments of the program, including the training of almost 6000 learners in the state; the process and outcomes of creating the screening approach called the “cognitive health assessment”; strategies for attracting more learners; environmental factors improving or hindering uptake of the training and screening benefit; and the success in building a research consortium in two county public health systems (Los Angeles, San Francisco) to study the impact of screening programs there on the quality of care provided to patients who screened positive. The presentation will summarize the key learnings and challenges of standing up a statewide program to support diverse primary care practices in taking up a cognitive screening benefit for low‐income residents.

